# Supplementary material for: Machine Learning Accelerates Crystallization for Structure Determination
Source: Angew Chem Int Ed Engl. 2026 May 4;65(25):e1218503. doi: 10.1002/anie.1218503 (PMC13266958; doi:10.1002/anie.1218503)

checkCIF/PLATON report

Structure factors have been supplied for datablock(s) lcz007

THIS REPORT IS FOR GUIDANCE ONLY. IF USED AS PART OF A REVIEW PROCEDURE FOR PUBLICATION, IT SHOULD NOT REPLACE THE EXPERTISE OF AN EXPERIENCED CRYSTALLOGRAPHIC REFEREE.

No syntax errors found. CIF dictionary Interpreting this report

Datablock: lcz007

Bond precision:	C-C = 0.0199 A		Wavelength=1.54184
Cell:	a=18.24293(11)	b=9.33640(5)	c=39.6212(2)
	alpha=90	beta=90	gamma=90
Temperature:	100 K		

	Calculated	Reported
Volume	6748.41(6)	6748.42(6)
Space group	P n a 21	P n a 21
Hall group	P 2c -2n	P 2c -2n
Moiety formula	C24 H15 Ag3 F18 N6 O2 [+ solvent]	C15 H3 Ag3 F18 N6, C9 H12 O2
Sum formula	C24 H15 Ag3 F18 N6 O2 [+ solvent]	C24 H15 Ag3 F18 N6 O2
Mr	1085.03	1085.03
Dx,g cm-3	2.136	2.136
Z	8	8
Mu (mm-1)	15.100	15.100
F000	4160.0	4160.0
F000'	4180.35	
h,k,lmax	23,11,50	22,11,50
Nref	14476[7345]	12664
Tmin,Tmax	0.156,0.163	0.001,1.000
Tmin'	0.069	

```
Correction method= # Reported T Limits: Tmin=0.001 Tmax=1.000
AbsCorr = MULTI-SCAN

Data completeness= 1.72/0.87       Theta(max)= 78.359

R(reflections)= 0.0765( 12490)                     wR2(reflections)=
                                                   0.2045( 12664)
S = 1.084                 Npar= 910
```

The following ALERTS were generated. Each ALERT has the format
test-name_ALERT_alert-type_alert-level.
Click on the hyperlinks for more details of the test.

Alert level B

```
PLAT780_ALERT_1_B Coordinates do not Form a Properly Connected Set     Please Do !
```

Author Response: The alert is due to a large number of co-crystallized molecules in the which sometimes do not show as a connected set. This is acceptable from a crystallographic point of view.

Alert level C

```
PLAT090_ALERT_3_C Poor Data / Parameter Ratio (Zmax > 18) ........       7.79 Note
PLAT213_ALERT_2_C Atom F5              has ADP max/min Ratio .....        3.8 prolat
PLAT213_ALERT_2_C Atom F12             has ADP max/min Ratio .....        3.1 prolat
PLAT213_ALERT_2_C Atom F13             has ADP max/min Ratio .....        3.4 oblate
PLAT213_ALERT_2_C Atom F01Q            has ADP max/min Ratio .....        3.2 prolat
PLAT213_ALERT_2_C Atom F029            has ADP max/min Ratio .....        3.6 oblate
PLAT213_ALERT_2_C Atom F01C            has ADP max/min Ratio .....        3.7 oblate
PLAT215_ALERT_3_C Disordered F9        has ADP max/min Ratio .....        3.7 Note
PLAT220_ALERT_2_C NonSolvent   Resd 1  C   Ueq(max)/Ueq(min) Range        4.3 Ratio
PLAT220_ALERT_2_C NonSolvent   Resd 1  F   Ueq(max)/Ueq(min) Range        3.4 Ratio
PLAT220_ALERT_2_C NonSolvent   Resd 2  C   Ueq(max)/Ueq(min) Range        3.8 Ratio
PLAT220_ALERT_2_C NonSolvent   Resd 2  F   Ueq(max)/Ueq(min) Range        3.2 Ratio
PLAT234_ALERT_4_C Large Hirshfeld Difference N01B     --N01R     .       0.16 Ang.
PLAT234_ALERT_4_C Large Hirshfeld Difference F2       --C01U     .       0.19 Ang.
PLAT234_ALERT_4_C Large Hirshfeld Difference N00X     --N00Y     .       0.16 Ang.
PLAT242_ALERT_2_C Low    'MainMol' Ueq as Compared to Neighbors of       N01B Check
PLAT242_ALERT_2_C Low    'MainMol' Ueq as Compared to Neighbors of       C00Z Check
PLAT342_ALERT_3_C Low Bond Precision on  C-C Bonds ...............    0.01988 Ang.
PLAT911_ALERT_3_C Missing FCF Refl Between Thmin & STh/L=    0.600          7 Report
                4  1  0,   8  4  0,  13  9  0,   2  0  2,   0  0  8,  15  1 34,
               14  0 36,
PLAT971_ALERT_2_C Check Calcd Resid. Dens.  1.62Ang From C00S            1.70 eA-3
PLAT971_ALERT_2_C Check Calcd Resid. Dens.  1.26Ang From F4              1.61 eA-3
PLAT972_ALERT_2_C Check Calcd Resid. Dens.  0.51Ang From N00I           -1.51 eA-3
PLAT977_ALERT_2_C Check Negative Difference Density on H01J      .      -0.34 eA-3
PLAT977_ALERT_2_C Check Negative Difference Density on H02B      .      -0.48 eA-3
PLAT977_ALERT_2_C Check Negative Difference Density on H33B      .      -0.31 eA-3
```

Alert level G

```
PLAT002_ALERT_2_G Number of Distance or Angle Restraints on AtSite         59 Note
PLAT003_ALERT_2_G Number of Uiso or U(i,j)  Restrained non-H-Atoms         48 Report
PLAT004_ALERT_5_G Polymeric Structure Found with Maximum Dimension          1 Info
PLAT042_ALERT_1_G Calc. and Reported MoietyFormula Strings  Differ     Please Check
              Calc: C24 H15 Ag3 F18 N6 O2
              Rep.: C15 H3 Ag3 F18 N6, C9 H12 O2
PLAT072_ALERT_2_G SHELXL First  Parameter in WGHT  Unusually Large       0.14 Report
PLAT083_ALERT_2_G SHELXL Second Parameter in WGHT  Unusually Large      60.75 Why ?
PLAT142_ALERT_4_G s.u. on b - Axis Small or Missing .............    0.00005 Ang.
PLAT143_ALERT_4_G s.u. on c - Axis Small or Missing .............    0.00020 Ang.
PLAT171_ALERT_4_G The CIF-Embedded .res File Contains EADP Records          4 Report
PLAT172_ALERT_4_G The CIF-Embedded .res File Contains DFIX Records         48 Report
PLAT176_ALERT_4_G The CIF-Embedded .res File Contains SADI Records          3 Report
PLAT177_ALERT_4_G The CIF-Embedded .res File Contains DELU Records         17 Report
PLAT178_ALERT_4_G The CIF-Embedded .res File Contains SIMU Records         15 Report
PLAT186_ALERT_4_G The CIF-Embedded .res File Contains ISOR Records         17 Report
PLAT188_ALERT_3_G A Non-default SIMU Restraint Value has been used     0.0100 Report
PLAT188_ALERT_3_G A Non-default SIMU Restraint Value has been used     0.0100 Report
PLAT188_ALERT_3_G A Non-default SIMU Restraint Value has been used     0.0100 Report
PLAT188_ALERT_3_G A Non-default SIMU Restraint Value has been used     0.0100 Report
PLAT188_ALERT_3_G A Non-default SIMU Restraint Value has been used     0.0100 Report
PLAT188_ALERT_3_G A Non-default SIMU Restraint Value has been used     0.0200 Report
PLAT188_ALERT_3_G A Non-default SIMU Restraint Value has been used     0.0200 Report
PLAT188_ALERT_3_G A Non-default SIMU Restraint Value has been used     0.0200 Report
PLAT192_ALERT_3_G A Non-default DELU Restraint Value for SecondPar     0.0200 Report
PLAT192_ALERT_3_G A Non-default DELU Restraint Value for SecondPar     0.0200 Report
PLAT242_ALERT_2_G Low    'MainMol' Ueq as Compared to Neighbors of        C18 Check
PLAT242_ALERT_2_G Low    'MainMol' Ueq as Compared to Neighbors of       C01I Check
PLAT242_ALERT_2_G Low    'MainMol' Ueq as Compared to Neighbors of       C01P Check
PLAT242_ALERT_2_G Low    'MainMol' Ueq as Compared to Neighbors of       C025 Check
PLAT242_ALERT_2_G Low    'MainMol' Ueq as Compared to Neighbors of       C02E Check
PLAT242_ALERT_2_G Low    'MainMol' Ueq as Compared to Neighbors of       C02Z Check
PLAT242_ALERT_2_G Low    'MainMol' Ueq as Compared to Neighbors of       C01J Check
PLAT242_ALERT_2_G Low    'MainMol' Ueq as Compared to Neighbors of       C01U Check
PLAT242_ALERT_2_G Low    'MainMol' Ueq as Compared to Neighbors of       C023 Check
PLAT242_ALERT_2_G Low    'MainMol' Ueq as Compared to Neighbors of       C02M Check
PLAT242_ALERT_2_G Low    'MainMol' Ueq as Compared to Neighbors of       C02Q Check
PLAT242_ALERT_2_G Low    'MainMol' Ueq as Compared to Neighbors of       C02S Check
PLAT299_ALERT_4_G Atom Site Occupancy Constrained at ............         0.5 Check
              F1      F1A     F3      F3A     F9      F9A
PLAT301_ALERT_3_G Main Residue  Disorder ..............(Resd    1)         6% Note
PLAT434_ALERT_2_G Short Inter HL..HL Contact F00L     ..F00R     .       2.81 Ang.
                                             -1/2+x,1/2-y,z  =      3_455 Check
PLAT605_ALERT_4_G Largest Solvent Accessible VOID in the Structure         14 A**3
PLAT720_ALERT_4_G Number of Unusual/Non-Standard Labels ..........        115 Note
              Ag01    Ag02    Ag03    Ag04    Ag05    Ag06    O007    F00A
              F00D    O00E    F00F    O00G    F00H    F00J    F00K    F00L
              F00M    F00N    F00Q    F00R    C00S    F00T    N00U    C01E
              C00O    H00O    C00W    N00I    F00V    N00X    N00Y    C02I
              C02L    H02L    C01V    C00Z    N010    N00P    C018    C016
              H016    C01M    C011    H01C    H01D    H01E    C012    H012
              C017    N00C    N008    C022    C014    H01F    H01G    H01H
              C015    O019    F01C    F01D    C01F    C01G    H01I    F01H
              C01I    C01J    C01K    H01A    H01B    C01L    C024    H024
              C01T    N009    N00B    C01N    H01J    H01K    C01O    C01P
              F01Q    F01S    C01U    C01W    F01X    F021    C023    C025
```

```
              F026    C028    F029    F02B    C02C    C02E    C02G    H02G
              C02M    C02O    H02A    H02B    H02C    C02Q    C02S    F02T
              C02U    H02D    H02E    H02H    C02V    C02F    H02F    C02A
              N01B    N01R    C02Z
PLAT802_ALERT_4_G CIF Input Record(s) with more than 80 Characters         1 Info
PLAT860_ALERT_3_G Number of Least-Squares Restraints ............        422 Note
PLAT868_ALERT_4_G ALERTS Due to the Use of _smtbx_masks Suppressed         ! Info
PLAT883_ALERT_1_G Absent Datum for _atom_sites_solution_primary ..     Please Do !
PLAT910_ALERT_3_G Missing FCF Reflection(s) Below Theta(Min)[Deg]=      4.85 Note
                0  0  2,   0  0  4,
PLAT912_ALERT_4_G Missing # of FCF Reflections Above STh/L=  0.600       243 Note
PLAT913_ALERT_3_G Missing # of Very Strong Reflections in FCF ....         2 Note
                4  1  0,   2  0  2,
PLAT915_ALERT_3_G No Flack x Check Done: Low Friedel Pair Coverage        78 %
PLAT941_ALERT_3_G Average HKL Measurement Multiplicity ..........        4.1 Low
PLAT969_ALERT_5_G The 'Henn et al.' R-Factor-gap value ..........      6.340 Note
              Predicted wR2: Based on SigI**2  3.23 or SHELX Weight 18.86
PLAT978_ALERT_2_G Number C-C Bonds with Positive Residual Density.         0 Info
```

```
   0 ALERT level A = Most likely a serious problem - resolve or explain
   1 ALERT level B = A potentially serious problem, consider carefully
  25 ALERT level C = Check. Ensure it is not caused by an omission or oversight
  52 ALERT level G = General information/check it is not something unexpected

   3 ALERT type 1 CIF construction/syntax error, inconsistent or missing data
  36 ALERT type 2 Indicator that the structure model may be wrong or deficient
  20 ALERT type 3 Indicator that the structure quality may be low
  17 ALERT type 4 Improvement, methodology, query or suggestion
   2 ALERT type 5 Informative message, check
```

Validation response form

Please find below a validation response form (VRF) that can be filled in and pasted into your CIF.

```
# start Validation Reply Form
_vrf_PLAT090_lcz007
;
PROBLEM: Poor Data / Parameter Ratio (Zmax > 18) ........       7.79 Note
RESPONSE: ...
;
_vrf_PLAT213_lcz007
;
PROBLEM: Atom F5              has ADP max/min Ratio .....        3.8 prolat
RESPONSE: ...
;
_vrf_PLAT215_lcz007
;
PROBLEM: Disordered F9        has ADP max/min Ratio .....        3.7 Note
RESPONSE: ...
;
_vrf_PLAT220_lcz007
;
PROBLEM: NonSolvent   Resd 1  C   Ueq(max)/Ueq(min) Range        4.3 Ratio
RESPONSE: ...
;
```

```
_vrf_PLAT234_lcz007
;
PROBLEM: Large Hirshfeld Difference N01B     --N01R     .       0.16 Ang.
RESPONSE: ...
;
_vrf_PLAT242_lcz007
;
PROBLEM: Low    'MainMol' Ueq as Compared to Neighbors of       N01B Check
RESPONSE: ...
;
_vrf_PLAT342_lcz007
;
PROBLEM: Low Bond Precision on  C-C Bonds ...............    0.01988 Ang.
RESPONSE: ...
;
_vrf_PLAT911_lcz007
;
PROBLEM: Missing FCF Refl Between Thmin & STh/L=    0.600          7 Report
RESPONSE: ...
;
_vrf_PLAT971_lcz007
;
PROBLEM: Check Calcd Resid. Dens.  1.62Ang From C00S            1.70 eA-3
RESPONSE: ...
;
_vrf_PLAT972_lcz007
;
PROBLEM: Check Calcd Resid. Dens.  0.51Ang From N00I           -1.51 eA-3
RESPONSE: ...
;
_vrf_PLAT977_lcz007
;
PROBLEM: Check Negative Difference Density on H01J      .      -0.34 eA-3
RESPONSE: ...
;
# end Validation Reply Form
```

It is advisable to attempt to resolve as many as possible of the alerts in all categories. Often the minor alerts point to easily fixed oversights, errors and omissions in your CIF or refinement strategy, so attention to these fine details can be worthwhile. It is up to the individual to critically assess their own results and, if necessary, seek expert advice.

PLATON version of 17/09/2025; check.def file version of 17/09/2025

---

duplicate check

No duplication found

Datablock lcz007 - ellipsoid plot

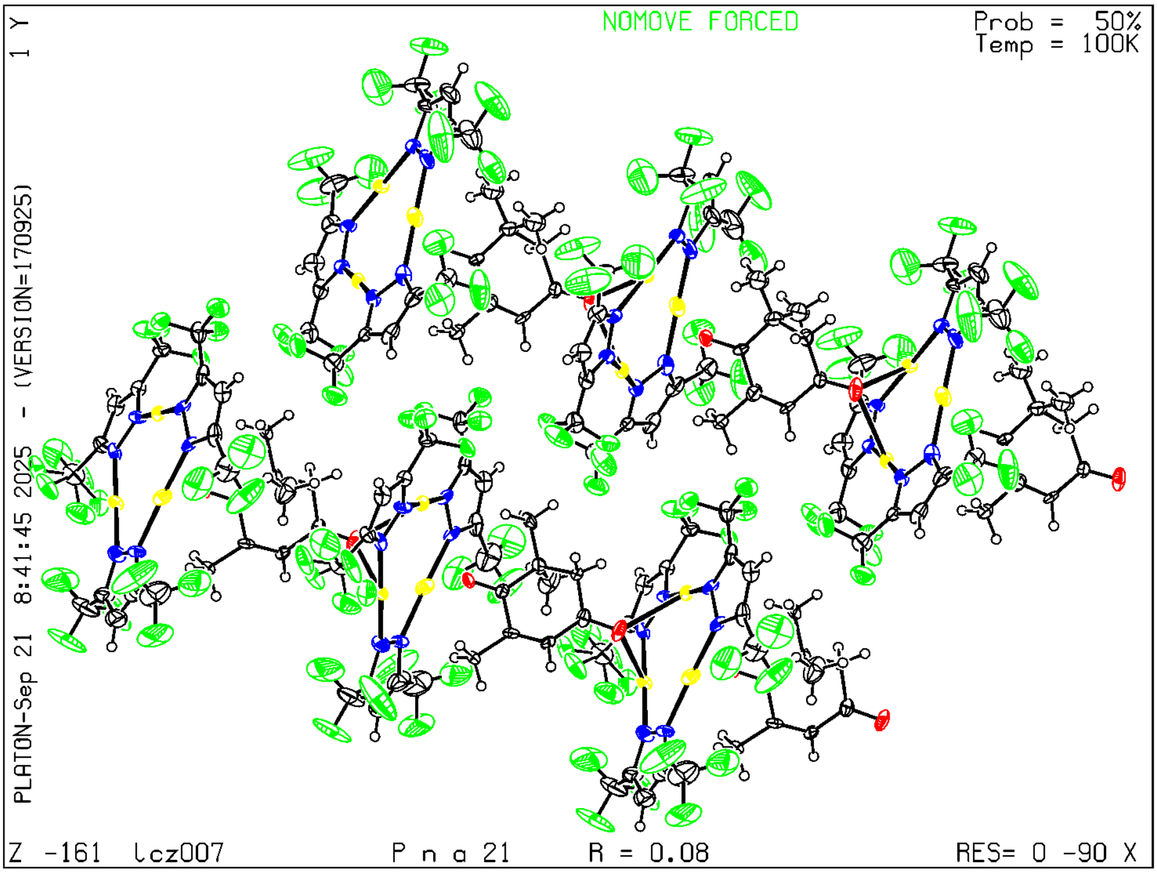

Supplement: Supplementary file 2 — Supporting File 2: anie72462‐sup‐0002‐DataFile.zip. [file ANIE-65-e1218503-s002.zip › supplementary_materials_crystallographic_data submit.zip!/cif_and_checkcif(1-114)/Ag3Pz3_7/Ag3Pz3_7_checkcif.pdf]
